# The perception of tooth whitening practices during and after orthodontic treatment: A survey of orthodontists

**DOI:** 10.4317/jced.57985

**Published:** 2021-06-01

**Authors:** Miguel-Fernando Niño, Stefanía Hernández-Viana, Felipe-Augusto Restrepo, Javier-Enrique Botero

**Affiliations:** 1 Facultad de Odontología, Universidad de Antioquia, Medellín Colombia

## Abstract

**Background:**

At present, there are limited studies on how tooth whitening procedures are applied in orthodontic patients. Therefore, the objective of this study was to assess the perception of tooth whitening practices during and after orthodontic treatment.

**Material and Methods:**

A survey of orthodontists in Medellín (Colombia) between January and October 2020 was carried out. The survey instrument was developed to obtain information regarding the perception and practices of tooth whitening during and after orthodontic treatment.

**Results:**

133 orthodontists with a mean age of 41,6 years old participated in the survey. Over 60% of participants reported >6 years of experience as orthodontist. The majority (99,2%) reported that their patients request tooth whitening. Of these, 71,2% refer the patient to another dental professional for the procedure while 28,8% administers it. More than half of orthodontists reported that their patients request dental whitening during orthodontic treatment. The majority (>90%) of orthodontists do not recommend tooth whitening during orthodontic treatment. Furthermore, >80% consider that tooth whitening is best recommended after brackets have been removed.

**Conclusions:**

Requests for tooth whitening from orthodontic patients is very frequent and most orthodontists favor the referral to another dental professional for the procedure. The majority do not recommend tooth whitening during orthodontic treatment and prefer waiting 1 to 3 months after brackets removal for the procedure.

** Key words:**Tooth whitening, orthodontic treatment, brackets.

## Introduction

The smile is a critical component of the attractiveness of the face that are important for human interactions. Therefore, the aesthetic self-perception of the smile is influenced by aspects of the gingival and tooth related aspects such as size, position, shape and color (1). Changes in the color of teeth are determined by intrinsic (characteristics of dentin and enamel) and extrinsic factors. Chromogens produced by organic components in the diet and cigarette smoking are the most common causes of extrinsic change in the color of teeth (2,3). It has been observed that change in the color of teeth can also be facilitated by orthodontic devices due to irregular accumulation of chromogens and plaque around brackets resulting in staining and white spot lesions (4,5).

Tooth whitening can be achieved by different protocols and the application during or after orthodontic treatment depends on the clinical case (6-8). Since the production of the first commercially available home-based tooth whitening system, the demand for whiter teeth has increased (9) This self-perceived necessity for whiter teeth has also been noticed by orthodontists. A national survey in the United States reported that 88,8% of orthodontists had patients that requested tooth whitening (10).

Limited evidence from clinical studies of tooth whitening performed during orthodontic treatment have been reported with satisfactory results regarding color improvement. 4 However, others consider that the presence of orthodontic brackets affects the effectivity of the whitening procedure under the brackets and consequently resulting in unpredictable improvements in color (11).

The most common recommendation for tooth whitening in orthodontic patients is after treatment has been completed and resulting changes in tooth color are clearly noticeable due to white spot lesions or extrinsic staining. A study reported that patients satisfaction improved after orthodontic treatment when tooth whitening was applied following brackets removal (12). Furthermore, over 60% of orthodontist refer their patients to other dental professional to manage tooth color changes. Only a small proportion of orthodontists (20-30%) provide cosmetic tooth whitening to their patients (2,10). Nonetheless, timing of whitening procedures after orthodontic treatment is not clearly defined by evidence-based studies and it remains a consideration of the clinician.

At present, there are limited studies on how tooth whitening procedures are applied in orthodontic patients. Therefore, the objective of this study was to assess the perception of tooth whitening practices during and after orthodontic treatment.

## Material and Methods

The study was a designed as a survey of orthodontists in Medellín (Colombia) between January and October 2020. The study was reviewed and approved by the institutional review board (44-2020).

-Instrument

The survey instrument was developed to obtain information regarding the perception and practices of tooth whitening during and after orthodontic treatment. For this purpose, demographic and frequency inquiries were designed as closed-ended questions. Reasons and further explanations for their preference of tooth whitening, referrals and whitening agent used were inquired in open-ended questions (supplemental material 1). The instrument was pilot evaluated in 10 orthodontists and any comments and corrections were made before it was applied. The final electronic questionnaire was produced in Google Forms and the personal information was protected by current data protection laws. Before the survey could be responded, the participant must have agreed with the informed consent, otherwise, the survey was finished and excluded.

-Sample

A sample of 132 surveys for analysis with 95% confidence and 5% error was calculated based on approximately 200 registered orthodontists in Medellín.

-Application of the survey

An invitation to participate in the survey was sent via text message (WhatsApp) to orthodontists registered in the database. The invitation was repeated every 2 months until the sample was completed.

-Data analysis

All responses were loaded in a spreadsheet for analysis. The demographic information is presented as the mean and 95% confidence interval (CI). All categorical responses are presented as frequency (%). The primary outcomes were (1) requests for tooth whitening from patients and (2) administration / referral for tooth whitening.

Open-ended responses were analyzed one by one. During the analysis, specific categories were identified, tabulated and the frequencies calculated.

## Results

**Figure 1:**
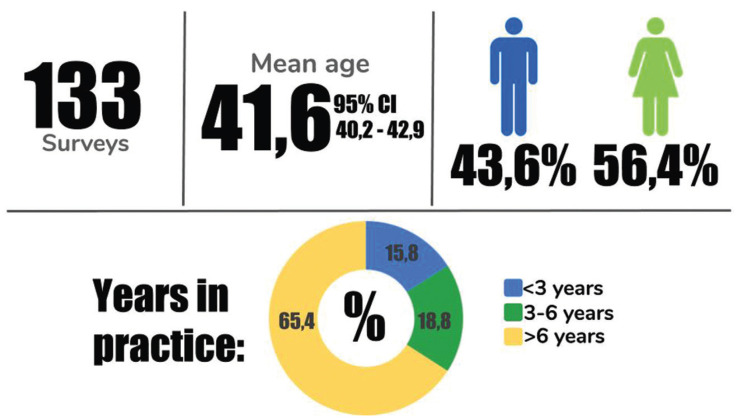
Demographic description of the sample.

Figure 1 shows the general characteristics of the study sample. One hundred and thirty-three surveys were completed and included for analysis from the 237 invitations sent. Therefore, the response rate was 56,1%. Participants in the survey were orthodontists with a mean age of 41,6 (95% CI 40,2 - 42,9) years old. The distribution of male and female participants was almost balanced with more female orthodontists included (56,4%). Over 60% of participants reported >6 years of experience as orthodontist.

The great majority (99,2%) of orthodontists reported that their patients request tooth whitening. Of these, 71,2% refer the patient to another dental professional for the procedure while 28,8% administer the procedure to their patients. More than half of orthodontists reported that their patients request tooth whitening during orthodontic treatment, whether they refer the patient (72,3%) or perform the procedure (60,5%). The majority (>90%) of orthodontists do not recommend tooth whitening during orthodontic treatment. Furthermore, >80% consider that tooth whitening is best recommended after brackets have been removed (Table 1).

**Table 1:**
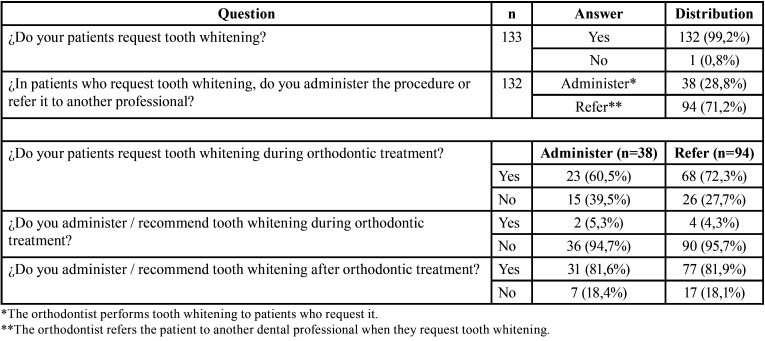
Distribution of orthodontists that administer or refer the procedure of tooth whitening during or after orthodontic treatment.

**Figure 2:**
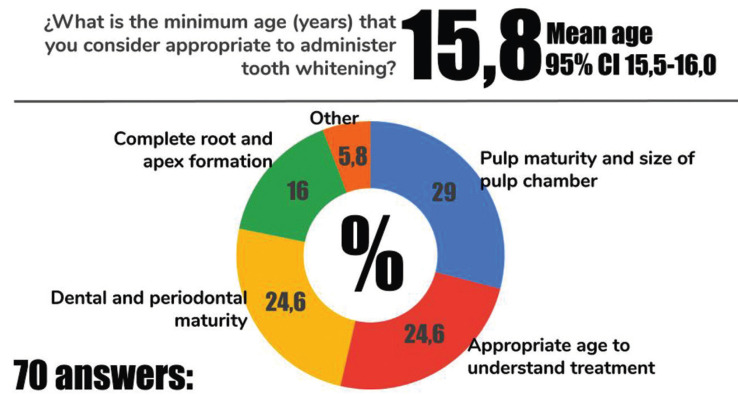
Minimum age and reasons for tooth whitening.

The minimum age recommendation for dental whitening and the corresponding reasons are presented in Figure 2. Orthodontists consider that the appropriate age for a patient to receive tooth whitening is around 15 years old (15,8; 95% CI 15,5-16,0). Pulp maturity (29%), appropriate age for comprehension of the treatment (24,6%) and dental and periodontal maturity (24,6%) were the most frequent reasons that justified the minimum age for tooth whitening.

**Figure 3:**
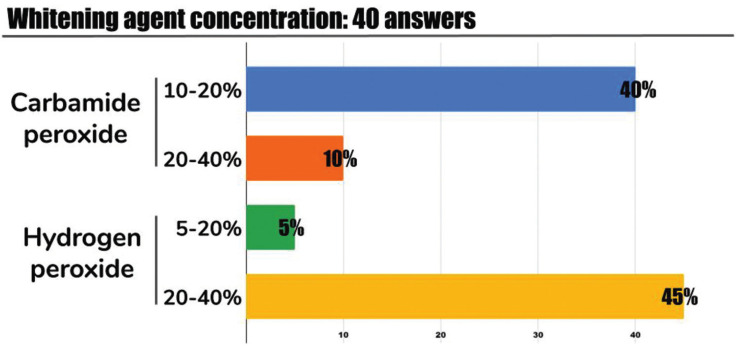
Distribution of whitening agents used.

The concentration and whitening agent used by orthodontists is depicted in Figure 3. Orthodontists who perform tooth whitening in their patients, whether during or after orthodontic treatment, use carbamide peroxide or hydrogen peroxide. Nonetheless, hydrogen peroxide in concentrations ranging 20-40% were most frequently used followed by carbamide peroxide in 10-20% concentration.

**Figure 4:**
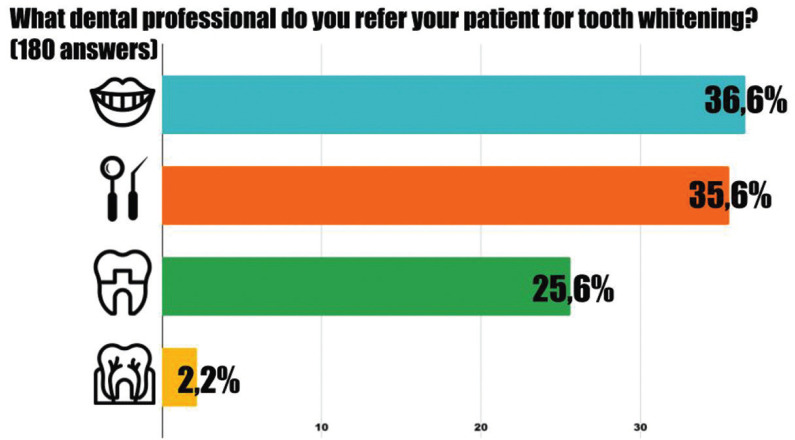
Distribution of the referral of patients. Top to bottom: dental aesthetic specialist, general dentist, prosthodontist, and endodontist.

Orthodontists who refer their patients for dental whitening were asked what dental specialty was of their preference (Fig. 4). The specialist in dental aesthetics was the most frequent (36,6%), followed by general dentists (35,6%) and prosthodontists (25,6%). Few orthodontists (2,2%) refer their patients to endodontists for tooth whitening.

Half of the participating orthodontists consider that after brackets removal, a period of 1 to 3 months should be waited before tooth whitening can be performed while 39,6% consider that this period should be greater than 3 months (Table 2). Periodontal reasons (35,6%) were the most frequent justification for this waiting period followed by pulp stability (18,7%) and factors related to the quality of the enamel (14,5%).

**Table 2:**
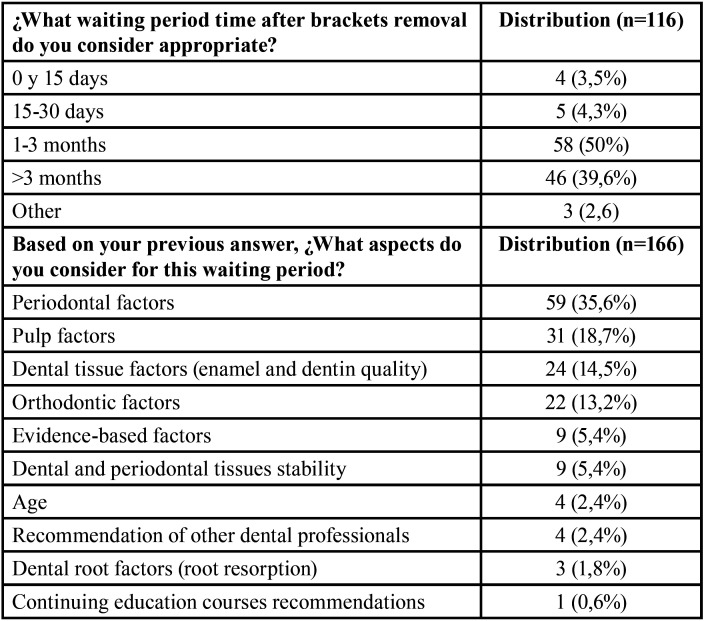
Waiting time period after brackets removal for tooth whitening.

## Discussion

This study assessed the perception of tooth whitening practices during or after orthodontic treatment by surveying orthodontists. The response rate was 56%, which is comparable to the study by Thickett and Cobourne (2) and Slack *et al*. (10) which obtained a 54% and 34% response rate, respectively. However, these differences may be due by the size of the sampling population frame. Slack *et al*. (10) conducted a nationwide survey with over 9000 orthodontists while our study focused only in one location. In contrast, Thickett and Cobourne 2 sent the questionnaires to a little over 1000 registered specialists and general dental professionals with interest in orthodontics. Another difference that may have accounted for this difference was the invitation strategy. Whereas Slack *et al*. (10) used e-mail and physical mail, we contacted orthodontists by means of text messages. Although the use of smartphones these days lets people to check their e-mails, people are more focused on social media and text messaging apps (WhatsApp). This allowed us to reach each participant more closely. Now regarding the response rate, it is possible that the 44% of non-respondents have no interest or do not perform tooth whitening in their practices.

From the results of our study, it can be observed that requests for tooth whitening from orthodontic patients are high and have possibly increased over time. In the year 2009 Thickett *et al*. (2) in the United Kingdom reported 92% requests and in 2013, Slack *et al*. (10) in the United States reported 88,8% requests. We found that 99,2% of the surveyed orthodontist have had requests for tooth whitening from their patients and this suggest an increasing trend (9). However, more orthodontists (71,2%) refer their patients to other dental professional for the procedure while only 28,8% perform the procedure. Similar results were obtained by Thickett and Cobourne (2) (refer 76% and 23% perform the procedure). In contrast, more orthodontists in the US perform (33%) tooth whitening to their patients and 66% refer the patient (10). Overall, orthodontists receive a high demand for tooth whitening but favor the referral of the patient for such procedure. Furthermore, the preferred dental professional for referrals were specialist in dental aesthetics (36,6%) and this may reflect the aesthetic impact that whiter and straight teeth have on patient satisfaction.

One important aspect that we assessed in contrast to the previous studies (2,10), was the perception of tooth whitening during and after orthodontic treatment. While 60-70% of orthodontists encounter requests for tooth whitening during orthodontic treatment, the great majority (>90%) do not recommend it. Instead, most orthodontists (80%) recommend the procedure after brackets have been removed and with a waiting period of at least 1 month (50%). This last recommendation is based primarily on conditions of the periodontium, pulp and enamel. Studies found that significant changes in blood flow of the pulp, plaque control and remineralization of the enamel occur one month after brackets debonding (13-15). Pulp tissue injuries could occur with high orthodontic forces that need time for repair (16). Nonetheless, a systematic review concluded that due to a lack of high-quality studies there is no conclusive scientific evidence for a relation between force level and pulp tissue reaction and the results of studies were contradictory (17). The only clinical study that determined how long it takes for the pulp to be completely normal after orthodontic treatment shows that sensibility tests are altered during orthodontic treatment and return to normal only after one year of retention (18). In this study, there was a diversity of reasons for this decision, reflecting a lack of consensus. Therefore, further clinical studies should provide evidence-based objective waiting periods to perform tooth whitening during or after orthodontic treatment.

This study did not attempt to discriminate between in-office and home applied whitening products. However, previous studies suggest that home whitening kits are preferred by orthodontists (70%) as compared to in-office procedures (2,10). Caution should be considered when recommending home whitening kits because of potential overuse (19) of the product by the patient that could result in adverse effects specially in the young population.

Orthodontists consider that the minimum appropriate age to receive tooth whitening is 15,8 years old and this is agreement with previous studies (2,10). Reasons for this minimum age included primarily pulp maturity, root development and appropriate age to comprehend the procedure. Nonetheless, the decision for tooth whitening in underage (<18 years old) patients should be discussed based on each clinical case and with the participation of the responsible parents.

## Conclusions

Requests for tooth whitening from orthodontic patients is very frequent and most orthodontists favor the referral to another dental professional for the procedure. The majority do not recommend tooth whitening during orthodontic treatment and prefer waiting 1 to 3 months after brackets removal for the procedure.
